# Supplementary material for: Dynalign II: common secondary structure prediction for RNA homologs with domain insertions
Source: Nucleic Acids Res. 2014 Nov 21;42(22):13939–48. doi: 10.1093/nar/gku1172 (PMC4267632; doi:10.1093/nar/gku1172)
Supplement: SUPPLEMENTARY DATA [file supp_gku1172_nar-02021-z-2014-File012.zip › manual/javadoc/serialized-form.html]

Serialized Form


JavaScript is disabled on your browser.


- Overview
- Package
- Class
- Tree
- Index
- Help

- Prev
- Next

- Frames
- No Frames

- All Classes

# Serialized Form

- ## Package RNAstructure\_java\_drawing.source.dialogs

  - ### Class RNAstructure\_java\_drawing.source.dialogs.ImageDialog extends JDialog implements Serializable

    serialVersionUID:
    :   20120802L

    - ### Serialized Fields

      - #### error

        ```
        String error
        ```

        The error message generated while building this panel, if any.
      - #### file

        ```
        String file
        ```

        The file that is drawn in some way on this panel.
      - #### label

        ```
        JLabel label
        ```

        The main label that describes the panel.
      - #### legendGrid

        ```
        JPanel legendGrid
        ```

        The panel that contains the legend grid.
      - #### mainPanel

        ```
        JPanel mainPanel
        ```

        The main panel that the drawing resides on.
      - #### maxBoundX

        ```
        double maxBoundX
        ```

        The maximum X bound of the image.
      - #### maxBoundY

        ```
        double maxBoundY
        ```

        The maximum Y bound of the image.
      - #### job

        ```
        PrinterJob job
        ```

        The job that prints out this panel
      - #### preferredDialogWidth

        ```
        int preferredDialogWidth
        ```

        The preferred dialog width.
      - #### scale

        ```
        double scale
        ```

        The scale at which the image is seen.
  - ### Class RNAstructure\_java\_drawing.source.dialogs.PlotDialog extends ImageDialog implements Serializable

    serialVersionUID:
    :   20120802L

    - ### Serialized Fields

      - #### backend

        ```
        DotPlotBackend backend
        ```

        The back end object that reads data for dot plots.
      - #### strand

        ```
        int strand
        ```

        The strand being drawn.
      - #### plotLength

        ```
        int plotLength
        ```

        The length of the plot.
  - ### Class RNAstructure\_java\_drawing.source.dialogs.StructureDialog extends ImageDialog implements Serializable

    serialVersionUID:
    :   20120802L

    - ### Serialized Fields

      - #### backend

        ```
        StructureBackend backend
        ```

        The back end object that reads data for structures.
      - #### data

        ```
        String[] data
        ```

        The array of data for a particular structure.
      - #### hasPairs

        ```
        boolean hasPairs
        ```

        Whether the current structure has pairs.
      - #### number

        ```
        int number
        ```

        The structure number viewed on this panel.
      - #### regex

        ```
        String[] regex
        ```

        The array of regexes used to pull data about a structure.
- ## Package RNAstructure\_java\_drawing.source.menus

  - ### Class RNAstructure\_java\_drawing.source.menus.AnnotationMenu extends RNAstructureMenu implements Serializable

    serialVersionUID:
    :   20120802L

    - ### Serialized Fields

      - #### dialog

        ```
        StructureDialog dialog
        ```

        The structure panel connected to this menu.
  - ### Class RNAstructure\_java\_drawing.source.menus.DotsMenu extends RNAstructureMenu implements Serializable

    serialVersionUID:
    :   20120615L

    - ### Serialized Fields

      - #### dialog

        ```
        PlotDialog dialog
        ```

        The structure dialog connected to this menu.
  - ### Class RNAstructure\_java\_drawing.source.menus.PlotMenu extends RNAstructureMenu implements Serializable

    serialVersionUID:
    :   20120615L

    - ### Serialized Fields

      - #### dialog

        ```
        PlotDialog dialog
        ```

        The plot dialog connected to this menu.
  - ### Class RNAstructure\_java\_drawing.source.menus.StructureMenu extends RNAstructureMenu implements Serializable

    serialVersionUID:
    :   20120802L

    - ### Serialized Fields

      - #### dialog

        ```
        StructureDialog dialog
        ```

        The structure dialog connected to this menu.
- ## Package RNAstructure\_java\_drawing.source.runners

  - ### Class RNAstructure\_java\_drawing.source.runners.PlotRunner extends RunnerTemplate implements Serializable

    serialVersionUID:
    :   20120802L
  - ### Class RNAstructure\_java\_drawing.source.runners.StructureRunner extends RunnerTemplate implements Serializable

    serialVersionUID:
    :   20120802L
- ## Package RNAstructure\_java\_interface.source.menus

  - ### Class RNAstructure\_java\_interface.source.menus.ConstraintsMenu extends RolloverMenu implements Serializable

    serialVersionUID:
    :   20120802L

    - ### Serialized Fields

      - #### backend

        ```
        RNAstructureBackendCalculator backend
        ```

        The back end calculator that's linked to this menu.
      - #### str

        ```
        int str
        ```

        The strand this constraints menu deals with.
  - ### Class RNAstructure\_java\_interface.source.menus.ConstraintsMenu.NucOrHelixDialog extends ValueSelectionDialog implements Serializable

    serialVersionUID:
    :   20120802L
  - ### Class RNAstructure\_java\_interface.source.menus.DynamicMenuBar extends JMenuBar implements Serializable

    serialVersionUID:
    :   20120802L
  - ### Class RNAstructure\_java\_interface.source.menus.FileMenu extends RolloverMenu implements Serializable

    serialVersionUID:
    :   20120802L
  - ### Class RNAstructure\_java\_interface.source.menus.HelpMenu extends RolloverMenu implements Serializable

    serialVersionUID:
    :   20120802L
  - ### Class RNAstructure\_java\_interface.source.menus.NucleicAcidMenu extends RolloverMenu implements Serializable

    serialVersionUID:
    :   20120802L
  - ### Class RNAstructure\_java\_interface.source.menus.RolloverMenu extends RNAstructureMenu implements Serializable

    serialVersionUID:
    :   20120802L
  - ### Class RNAstructure\_java\_interface.source.menus.RolloverMenu.RolloverListener extends Object implements Serializable

    serialVersionUID:
    :   20120802L

    - ### Serialized Fields

      - #### rollover

        ```
        String rollover
        ```

        The rollover text for this listener.
- ## Package RNAstructure\_java\_interface.source.startup

  - ### Class RNAstructure\_java\_interface.source.startup.ApplicationRootFrame extends JFrame implements Serializable

    serialVersionUID:
    :   20120802L

    - ### Serialized Fields

      - #### bar

        ```
        JToolBar bar
        ```

        The main RNAstructure toolbar.
  - ### Class RNAstructure\_java\_interface.source.startup.RNAstructure extends Object implements Serializable

    serialVersionUID:
    :   20120802L
- ## Package RNAstructure\_java\_interface.source.windows

  - ### Class RNAstructure\_java\_interface.source.windows.DrawingWindow extends InternalWindow implements Serializable

    serialVersionUID:
    :   20120802L

    - ### Serialized Fields

      - #### dialog

        ```
        ImageDialog dialog
        ```

        The image dialog connected to this window.
      - #### error

        ```
        boolean error
        ```

        True if an error occurred building this window, false if not.
      - #### strand

        ```
        int strand
        ```

        The strand being drawn.
  - ### Class RNAstructure\_java\_interface.source.windows.DynalignFoldWindow extends ModuleWindow implements Serializable

    serialVersionUID:
    :   20120802L
  - ### Class RNAstructure\_java\_interface.source.windows.DynalignRefoldWindow extends ModuleWindow implements Serializable

    serialVersionUID:
    :   20120802L
  - ### Class RNAstructure\_java\_interface.source.windows.Efn2Window extends ModuleWindow implements Serializable

    serialVersionUID:
    :   20120802L
  - ### Class RNAstructure\_java\_interface.source.windows.FoldDoubleWindow extends ModuleWindow implements Serializable

    serialVersionUID:
    :   20120802L
  - ### Class RNAstructure\_java\_interface.source.windows.FoldSingleWindow extends ModuleWindow implements Serializable

    serialVersionUID:
    :   20120802L
  - ### Class RNAstructure\_java\_interface.source.windows.FoldSuboptimalWindow extends ModuleWindow implements Serializable

    serialVersionUID:
    :   20120802L
  - ### Class RNAstructure\_java\_interface.source.windows.InternalWindow extends JInternalFrame implements Serializable

    serialVersionUID:
    :   20120802L

    - ### Serialized Fields

      - #### backend

        ```
        RNAstructureBackendCalculator backend
        ```

        The back end calculator that's linked to this window.
      - #### dialogHandler

        ```
        SimpleDialogHandler dialogHandler
        ```

        A dialog handler to show any simple dialog messages that pop up from
        this window.
      - #### menuBar

        ```
        DynamicMenuBar menuBar
        ```

        The menu bar that's attached to this window.
  - ### Class RNAstructure\_java\_interface.source.windows.MaxExpectWindow extends ModuleWindow implements Serializable

    serialVersionUID:
    :   20120802L
  - ### Class RNAstructure\_java\_interface.source.windows.ModuleWindow extends InternalWindow implements Serializable

    serialVersionUID:
    :   20120802L

    - ### Serialized Fields

      - #### constraints

        ```
        GridBagConstraints constraints
        ```

        The GridBagConstraints for this window.
      - #### layout

        ```
        GridBagLayout layout
        ```

        The layout for this window.
      - #### isRNA

        ```
        boolean isRNA
        ```

        A boolean, true if the calculation handles RNA, false if not.
      - #### progressBar

        ```
        JDialog progressBar
        ```

        The progress bar dialog for this window.
  - ### Class RNAstructure\_java\_interface.source.windows.MultilignWindow extends MultiWindow implements Serializable

    serialVersionUID:
    :   20120802L
  - ### Class RNAstructure\_java\_interface.source.windows.MultiWindow extends ModuleWindow implements Serializable

    serialVersionUID:
    :   20120802L

    - ### Serialized Fields

      - #### columnWidth

        ```
        int columnWidth
        ```

        The width of each column of the window (input controls in column 1,
        sequence set in column 2).
      - #### imageDialogs

        ```
        ArrayList<E> imageDialogs
        ```

        The list of drawing windows shown after the calculation is done.
      - #### options

        ```
        Box options
        ```

        The box that holds a particular window's unique options.
      - #### scroll

        ```
        ScrollerPane scroll
        ```

        The scroll pane holding the sequence set.
  - ### Class RNAstructure\_java\_interface.source.windows.OligoScreenWindow extends ModuleWindow implements Serializable

    serialVersionUID:
    :   20120802L
  - ### Class RNAstructure\_java\_interface.source.windows.OligoWalkWindow extends ModuleWindow implements Serializable

    serialVersionUID:
    :   20120802L

    - ### Serialized Fields

      - #### isInputWindow

        ```
        boolean isInputWindow
        ```

        A boolean that tells whether this window is an input window or not.
      - #### panelInput

        ```
        JPanel panelInput
        ```
      - #### panelResults

        ```
        JPanel panelResults
        ```

        The box that holds the results.
  - ### Class RNAstructure\_java\_interface.source.windows.OligoWalkWindow.OligoSelectionDialog extends ValueSelectionDialog implements Serializable

    serialVersionUID:
    :   20120802L
  - ### Class RNAstructure\_java\_interface.source.windows.OligoWalkWindow.SpinnerPanel extends JPanel implements Serializable

    serialVersionUID:
    :   20120802L

    - ### Serialized Fields

      - #### spinner

        ```
        JSpinner spinner
        ```

        The spinner in this panel.
  - ### Class RNAstructure\_java\_interface.source.windows.OligoWalkWindow.StringComboBox extends JComboBox implements Serializable

    serialVersionUID:
    :   20120802L
  - ### Class RNAstructure\_java\_interface.source.windows.PartitionDoubleWindow extends ModuleWindow implements Serializable

    serialVersionUID:
    :   20120802L
  - ### Class RNAstructure\_java\_interface.source.windows.PartitionSingleWindow extends ModuleWindow implements Serializable

    serialVersionUID:
    :   20120802L
  - ### Class RNAstructure\_java\_interface.source.windows.ProbKnotWindow extends ModuleWindow implements Serializable

    serialVersionUID:
    :   20120802L
  - ### Class RNAstructure\_java\_interface.source.windows.PseudoknotWindow extends ModuleWindow implements Serializable

    serialVersionUID:
    :   20120802L
  - ### Class RNAstructure\_java\_interface.source.windows.RefoldWindow extends ModuleWindow implements Serializable

    serialVersionUID:
    :   20120802L
  - ### Class RNAstructure\_java\_interface.source.windows.SequenceDisplayWindow extends InternalWindow implements Serializable

    serialVersionUID:
    :   20120802L

    - ### Serialized Fields

      - #### areas

        ```
        ArrayList<E> areas
        ```

        The array of text areas in this window.
      - #### edited

        ```
        boolean edited
        ```

        A boolean, true if the sequence has been edited, false if not.
      - #### position

        ```
        int position
        ```

        The position at which reading is occurring.
      - #### readButton

        ```
        JButton readButton
        ```

        The button which can be clicked to start or stop reading.
      - #### reading

        ```
        boolean reading
        ```

        A boolean, true if reading while typing is active, false if not.
      - #### timer

        ```
        Timer timer
        ```

        The timer that aids in reading nucleotides in rhythm.
  - ### Class RNAstructure\_java\_interface.source.windows.StochasticWindow extends ModuleWindow implements Serializable

    serialVersionUID:
    :   20120802L
  - ### Class RNAstructure\_java\_interface.source.windows.TurboFoldWindow extends MultiWindow implements Serializable

    serialVersionUID:
    :   20120802L

    - ### Serialized Fields

      - #### cards

        ```
        CardLayout cards
        ```

        The layout that holds the dynamic options applicable to a mode.
      - #### specificPanel

        ```
        JPanel specificPanel
        ```

        The panel that holds the dynamic options applicable to a mode.
- ## Package RNAstructure\_java\_utilities

  - ### Class RNAstructure\_java\_utilities.BorderBuilder extends Object implements Serializable

    serialVersionUID:
    :   20120802L
  - ### Class RNAstructure\_java\_utilities.BorderedButtonPanel extends JPanel implements Serializable

    serialVersionUID:
    :   20120802L
  - ### Class RNAstructure\_java\_utilities.FieldPanel extends JPanel implements Serializable

    serialVersionUID:
    :   20120802L

    - ### Serialized Fields

      - #### components

        ```
        JComponent[] components
        ```

        The components on this panel (buttons or labels).
      - #### specifiedPanelWidth

        ```
        int specifiedPanelWidth
        ```

        The specified width of the panel.
      - #### textFields

        ```
        JTextField[] textFields
        ```

        The array of text fields.
  - ### Class RNAstructure\_java\_utilities.FieldPanel.FilePanel extends FieldPanel implements Serializable

    serialVersionUID:
    :   20120802L
  - ### Class RNAstructure\_java\_utilities.FieldPanel.NumberPanel extends FieldPanel implements Serializable

    serialVersionUID:
    :   20120802L
  - ### Class RNAstructure\_java\_utilities.FileChooser extends Object implements Serializable

    serialVersionUID:
    :   20120802L

    - ### Serialized Fields

      - #### defaultFile

        ```
        String defaultFile
        ```

        The default file name for this file chooser, if necessary.
      - #### selectorType

        ```
        FileChooser.Types selectorType
        ```

        The type of file type selector.
  - ### Class RNAstructure\_java\_utilities.FileField extends JTextField implements Serializable

    serialVersionUID:
    :   20120802L
  - ### Class RNAstructure\_java\_utilities.HTMLCheckBox extends JCheckBox implements Serializable

    serialVersionUID:
    :   20120802L
  - ### Class RNAstructure\_java\_utilities.ImageGrabber extends Object implements Serializable

    serialVersionUID:
    :   20120802L
  - ### Class RNAstructure\_java\_utilities.MacChecker extends Object implements Serializable

    serialVersionUID:
    :   20120802L
  - ### Class RNAstructure\_java\_utilities.NumberField extends JTextField implements Serializable

    serialVersionUID:
    :   20120802L

    - ### Serialized Fields

      - #### initial

        ```
        Number initial
        ```

        The initial default value of the field.
      - #### maximum

        ```
        Number maximum
        ```

        The maximum value of the field.
      - #### minimum

        ```
        Number minimum
        ```

        The minimum value of the field.
  - ### Class RNAstructure\_java\_utilities.NumberField.DoubleField extends NumberField implements Serializable

    serialVersionUID:
    :   20120802L
  - ### Class RNAstructure\_java\_utilities.NumberField.FloatField extends NumberField implements Serializable

    serialVersionUID:
    :   20120802L
  - ### Class RNAstructure\_java\_utilities.NumberField.IntegerField extends NumberField implements Serializable

    serialVersionUID:
    :   20120802L
  - ### Class RNAstructure\_java\_utilities.RadioButtonPanel extends JPanel implements Serializable

    serialVersionUID:
    :   20120802L

    - ### Serialized Fields

      - #### group

        ```
        ButtonGroup group
        ```

        The button group connected to this panel.
      - #### group2

        ```
        ArrayList<E> group2
        ```

        The button group, as a list, for ease of access to each.
      - #### names

        ```
        String[] names
        ```

        The names of the buttons in the group.
  - ### Class RNAstructure\_java\_utilities.RNAstructureMenu extends JMenu implements Serializable

    serialVersionUID:
    :   20120802L
  - ### Class RNAstructure\_java\_utilities.ScrollerPane extends JScrollPane implements Serializable

    serialVersionUID:
    :   20120802L
  - ### Class RNAstructure\_java\_utilities.SimpleDialogHandler extends Object implements Serializable

    serialVersionUID:
    :   20120802L
  - ### Class RNAstructure\_java\_utilities.ValueSelectionDialog extends JDialog implements Serializable

    serialVersionUID:
    :   20120802L

    - ### Serialized Fields

      - #### fields

        ```
        NumberField[] fields
        ```

        The array of number fields this dialog holds.

- Overview
- Package
- Class
- Tree
- Index
- Help

- Prev
- Next

- Frames
- No Frames

- All Classes
